# Dairy Producers Who Market Their Surplus Progeny as Calves Use Germplasm With Slightly Lighter and Less-Conformed Carcasses Than Producers Who Rear Their Surplus Progeny Beyond Weaning

**DOI:** 10.3389/fvets.2021.731894

**Published:** 2021-10-13

**Authors:** Donagh P. Berry, Siobhan R. Ring

**Affiliations:** ^1^Teagasc, Animal and Grassland Research and Innovation Centre, Fermoy, Ireland; ^2^Irish Cattle Breeding Federation, Bandon, Ireland

**Keywords:** sire selection, beef-on-dairy, prime, carcass, veal

## Abstract

Understanding dairy producer mindset in service sire selection can provide useful information for different junctures along the commercial and extension animal breeding chain. It can aid the targeted marketing of bulls based on farm production systems but also provide useful information for delivering bespoke extension services. The objective of the present study was to examine if differences exist among dairy producers in their choice of dairy and beef service sires depending on the life stage at which the surplus progeny generated from such matings exit the dairy farm. This was predominantly based on evaluating the breed of beef sires used but also their genetic merit for calving difficulty and carcass traits, namely, carcass weight, conformation, and fat score; differences in genetic merit among dairy sires as well as among the dairy cows themselves were also considered. The objective was accomplished through the cross-sectional analyses of progeny fate data from 1,092,403 progeny born in 4,117 Irish dairy herds. Herd-years were categorized into one of four systems based on when the surplus progeny exited the dairy farm: (1) calves sold <70 days of age, (2) cattle sold as yearlings between 250 and 450 days of age, (3) prime cattle sold for finishing (slaughtered between 8 and 120 days of exiting the dairy farm), or (4) prime cattle sold for immediate slaughter (i.e., slaughtered within 7 days of exiting the dairy farm). The mean genetic merit of both the cows and service sires used across the four different systems was estimated using linear mixed models. Of the beef service sires used in herds that sold their surplus progeny as calves, their mean predicted transmitting ability for carcass weight and carcass conformation score was just 2.00 kg and 0.11 scores [scale of 1 (poor) to 15 (excellent)] inferior to the beef service sires used in herds that sold their surplus progeny as prime cattle for immediate slaughter. Similar trends, albeit of smaller magnitude, were evident when comparing the genetic merit of the dairy service sires used in those systems. Cows in herds that sold their surplus progeny as calves were genetically less likely to incur dystocia as well as to have lighter, less-conformed, and leaner carcasses than cows in herds that sold their surplus progeny post-weaning. Hence, results from the present study suggest that diversity in herd strategy regarding when surplus progeny exit the herd influences service sire selection choices in respect of genetic merit for dystocia and carcass attributes. That said, the biological difference based on the current pool of available service sires is small relative to the dairy producers that sell their surplus progeny as young calves; when expressed on a per standard deviation in genetic merit of the beef service sires used across all herds, the difference between extreme systems was, nonetheless, approximately half a standard deviation for carcass weight and conformation.

## Introduction

The choice of which bulls to use as service sires in a dairy herd is a topic of constant discussion and debate. Breeding objectives do, however, exist to aid producers in ranking candidate dairy (1) and beef (2) sires for use on dairy females; nonetheless, breeding objectives are one of the most contentious components of a breeding program, as they are conditional on the underlying parameters and assumptions specified in the calculation of the weighting factors. Tailored breeding objectives for individual herds through altering the emphasis on different suites of traits is facilitated by presenting sub-indexes of the overall breeding goal (1, 3, 4); customized selection indexes (5) are another such approach. Irrespective, lists of many candidate sires ranked on a total merit index are published (www.icbf.com; www.uscdcb.com; www.dairynz.co.nz), affording an opportunity for producers to select from within. Understanding how the service sires chosen vary by the demographics of dairy producers can provide useful insights into their respective psyche. This, in turn, provides feedback to the breeders and marketers of bulls, as well as the researchers, as to the demands of the different segments of the sector. Understanding actual service sire usage can also help understand why historical genetic trends may differ from those predicted using selection index theory.

Given the growing use of beef on dairy globally (6, 7), the beef merit of progeny from the dairy herd is receiving ever-intensifying scrutiny (8). This may contribute to a greater consciousness among dairy producers of the beef merit of both the dairy and beef sires mated to dairy females, as well as the beef merit of the dairy females themselves. Many Irish (dairy) farms are fragmented consisting of several land parcels (9). Hence, many Irish dairy producers rear their surplus progeny on land not in the vicinity of the milking parlor. Some dairy producers sell their surplus progeny as calves, others may rear their surplus progeny until 6–14 months of age (e.g., before or after the first winter housing period), and others may rear their surplus progeny until they are (almost) ready for slaughter; a combination of systems is also common. Hence, this type of dataset provides a unique opportunity to explore (dairy and beef) sire selection depending on the fate of the eventual surplus calves; the results should be generalizable to other populations.

The hypothesis in the present study was that a dairy producer who rears (a large proportion of) the surplus progeny to yearlings or older may place a greater emphasis on the beef characteristics of both the dairy and beef service sires used relative to the dairy producers who sell such stock as calves; the same hypothesis could be true for cows residing in herds that rear surplus progeny to yearlings or older. The justification for said hypotheses is that dairy producers that rear their surplus cattle up until or close to slaughter are likely to reap the rewards from utilizing superior germplasm for carcass attributes. Testing of these hypotheses was undertaken using a cross-sectional analysis of the Irish national database by investigating the genetic merit for carcass and calving performance of the cows and service sires used in dairy herds depending on what stage of life the surplus progeny exited the dairy farm.

## Materials and Methods

Data used in the present study were extracted from the Irish Cattle Breeding Federation (http://www.icbf.com) database. All inter-location animal movements, including the herd of origin and destination, were available on 8,128,696 calves born in 14,019 Irish dairy herds between the years 2014 and 2020. Of particular interest in the present study was the genetic merit of the sires and dams for both calving performance and carcass traits. Therefore, the Irish national genetic evaluations for carcass and calving performance traits in the year immediately prior to the birth of the calf (i.e., the genetic evaluation available at the time of conception of the calf) were also available for all years; all genetic evaluations are expressed on the scale of predicted transmitting ability (PTA). The national genetic evaluations are undertaken across all breeds and re-based to the same across-breed base population, thus ensuring that the breed-specific PTAs are directly comparable (10–12). The statistical model, variance components, and base population did not change for any of these traits during the study period.

Calving difficulty in Ireland is recorded on a 4-point scale, as follows: (1) no assistance, (2) assistance provided with some calving difficulty, (3) assistance provided with considerable calving difficulty but without veterinary intervention, and (4) assistance provided with considerable calving difficulty resulting in veterinary intervention. Genetic evaluations for calving difficulty are generated from a multi-trait animal-dam linear mixed model, which also includes gestation length and perinatal mortality as correlated traits; the resulting PTAs for calving difficulty are transformed to a percentage of progeny expected to experience dystocia (i.e., score of 3 or 4). Gestation length per animal is calculated as the number of days between the last available service date and the subsequent calving date assuming it is between 271 and 300 days in length; the genetic evaluation for gestation length is based on a multi-trait linear animal mixed model.

PTAs for carcass weight, conformation, and fat cover in Ireland, all of which are of interest to the present study, are evaluated on both prime cattle and cows as correlated traits. Genetic evaluations for the three carcass traits (i.e., prime cattle and cows separately) are undertaken using a multi-trait model that also includes feed intake as well as live-weight measures at different age categories represented as different traits in the evaluation; only direct genetic effects are considered for the carcass traits in the national genetic evaluation. Carcass weight is recorded within 1 h after slaughter (following the removal of the head, hide, feet, legs, thoracic organs, abdominal organs, and internal fat) to measure the hot weight of the carcass, which is then multiplied by 0.98 to estimate the cold carcass weight. Carcass conformation and fat cover in Ireland are based on the EU beef carcass classification system (EUROP) for conformation and subcutaneous fat cover graded using video image analysis (13, 14); no S conformation class is used in Ireland. The 15-point conformation classification system attempts to describe the conformation of the animal based mainly on the round, back, and shoulder. A score of 1 reflects poor conformation, whereas a score of 15 reflects excellent conformation. Carcass fat score attempts to describe the fat cover on the outside of the carcass and in the thoracic cavity and is graded on a 15-point scale from 1 (low fat cover) to 15 (high fat cover).

Inter-location animal movement data were used to determine the eventual fate of the progeny born in a given dairy herd. Each animal was categorized based on its stage of life when it exited the dairy farm of birth. Categories of age considered when exiting the dairy farm were (1) calves that exited the dairy farm <70 days of age; (2) yearlings, where the animal exited the dairy farm between 150 and 450 days of age but was not slaughtered within 120 days of sale; (3) finishers, where the animal was not sold for immediate slaughtering but was slaughtered between 8 and 120 days of exiting the dairy farm, assuming it was >300 days of age when leaving the farm (and had never been a parent); and (4) slaughtered immediately from the farm where the number of days between exiting the farm and being slaughtered was ≤7 days but also where the animal was >400 days of age when slaughtered (and had never been a parent). Female progeny from dairy sires were not considered in the present study, as these would not normally be deemed to be surplus animals destined for meat production. The destination of the surplus animals is quite varied; some calves, particularly the dairy male calves, are exported predominantly to the Netherlands (15) for veal production, with the remainder being purchased by Irish beef producers for rearing (and often re-sale several months later). Many of the yearling animals and finisher animals are sold to Irish beef producers with only a small quantity (from dairy herds) exported.

The number of progeny in each life-stage category as a percentage of all progeny born in a given herd-year was determined; not considered in the denominator were the female progeny sired by dairy bulls and animals that died on farm. Based on the percentage of animals in each category, the system of marketing surplus progeny was defined for each herd-year. Where >66% of the surplus progeny born in a given herd-year were categorized into a single life-stage on exit, then that herd-year was assumed to adopt that system; this resulted in a considerable loss of data. Furthermore, because dairy producers can sometimes switch between production systems across years, the present study only focused on herds that were relatively consistent in what life stages they marketed their surplus progeny. Therefore, a herd had to have consistently been coded into the same system of selling surplus progeny for at least 4 of the 7 years (i.e., 66% of the years). Only herds generating between 50 and 300 surplus calves annually (i.e., excluding dairy heifers) were retained; and herd size was categorized as 50 to 99 surplus calves per year, 100–149 surplus calves per year, 150–199 surplus calves per year, 200–249 surplus calves per year, and 250–300 surplus calves per year.

Of the herd-years remaining after edits, a further 27% of animals were discarded, as their sire was unknown. Following all edits, data on 1,092,403 progeny from 610,261 dairy cows in 4,117 herds and 16,521 herd-years remained for analyses. The number of beef females, beef males, and dairy males included in the analysis was 240,568, 260,223, and 591,612, respectively.

### Statistical Analyses

Linear mixed models in ASReml (16) were used to quantify the least squares means for the carcass and calving performance PTAs of the service sires and dams of surplus progeny by herd system representing which stage of life the surplus progeny exited the dairy farm. The model fitted was


$$\begin{array}{l} \text{Y}=\text{herd\_size}+\text{dam\_parity}+\text{system}+\text{herd}\text{-year}+\text{ e} \end{array}$$

where Y is the dependent variable representing sire or dam (of progeny) genetic merit for each carcass or calving performance trait, herd_size is the categorization (*n* = 5) of the number of surplus calves born in a given year, dam_parity is the parity of the dam of the progeny in question, system is the predominant market of the progeny (i.e., calves, yearlings, finishers, and immediate slaughter) born in that herd-year, herd-year is the random effect of herd-year, and e is the random residual term. When the dependent variable was the PTA of the service sire, the analyses were undertaken for dairy-sired and beef-sired progeny separately; both datasets were combined when exploring any association between the life stage of sale of surplus progeny and dam PTA.

Also investigated, albeit just for the progeny sired by beef bulls, was the likelihood of the beef sire used being of a certain breed; the breeds investigated were Angus, Belgian Blue, Charolais, Hereford, Limousin, and Simmental, as these were the predominant beef breeds in the study and used in Ireland. The logit of the probability of a given breed of sire being used was modeled separately for each breed as a binary outcome using the model already described except that the binomial distribution of the error term was also accounted for. The overlap in artificial insemination (AI) sires used across the different systems was also quantified.

## Results

Of the 16,521 herd-years represented in the dataset, 14,567 (90%) sold their surplus progeny as calves with 705 (4%), 345 (2%), and 904 (5%) herd-years selling their surplus as yearlings, finishers, or immediately for slaughter, respectively; the percentage of animals in each system followed a similar trend with 979,847 (90%) progeny sold as calves, 36,423 (3%) sold as yearlings, 20,258 (2%) sold as finishers, and 52,875 (5%) sold for immediate slaughter. The mean herd size for each system did not differ dramatically, although the herds that sold the majority of their surplus progeny as calves were larger; the mean (standard deviation) herd size (i.e., number of surplus calves) for herds that sold their surplus progeny as calves, yearlings, finishers, and for immediate slaughter was 84 (35), 76 (28), 76 (26), and 75 (26), respectively.

The beef-sired animals in the edited dataset originated from 10,465 different sires, 951 of which were AI sires. Of the 951 AI sires, 76 (i.e., 8%) had progeny in all four systems. The number of AI beef sires in common between each pairwise combination of systems is in Table 1. Of the beef AI sires, 16–37% were common to each pair of systems; for example, of the 928 AI beef sires used by dairy producers that sold surplus progeny as either calves or yearlings, 223 of those sires had progeny in both systems. The extent of common sires across production systems, however, manifested itself as 82–90% of the progeny in each system having a sire in common with the other system. The dairy-sired males in the edited dataset originated from 9,550 different sires, 2,488 of which were AI sires. Of the 2,488 AI sires, 246 (i.e., 10%) had progeny in all four systems. The number of AI dairy sires in common between each pairwise combination of systems is in Table 1. Of the dairy AI sires, 17–44% were common across pairs of systems reflecting 67–84% of the progeny across two systems having a sire in common.

**Table 1:** Contingency table of the number of beef (lower diagonal) and dairy (upper diagonal) AI sires with progeny in each pairwise system comparison as a function, in parentheses, of the total number of AI sires used across both systems.

**System**	**Calves**	**Yearlings**	**Finishers**	**Slaughter**
Calves		679 (2,449)	400 (2,416)	702 (2,446)
Yearlings	223 (928)		323 (801)	445 (1,011)
Finishers	142 (913)	100 (294)		302 (842)
Slaughter	231 (923)	133 (360)	96 (301)	

*AI, artificial insemination*.

### Beef Service Sires Used

The mean genetic merit of beef service sires used in herds differing on when they marketed their surplus progeny is in Table 2. Herds that reared their surplus progeny to a finishing stage (i.e., slaughtered within 120 days of exiting the dairy herd of origin) used beef sires that were genetically prone to more dystocia, but with heavier and more conformed carcasses than herds that sold their surplus at younger life stages. With the exception of carcass fat, a difference (*p* < 0.05) in mean genetic merit always existed between the beef service sires chosen for use in herds that predominantly sold their surplus progeny as calves and those that sold their surplus progeny for finishing or immediate slaughter; the beef sires of surplus progeny sold as calves were genetically easier calving, had a shorter gestation, and had lighter, less-conformed carcasses. Based on the standard deviation in PTAs for the beef service sires used across all herds, the difference in mean PTA for carcass weight and carcass conformation score between the beef service sires used in herds that sold their surplus progeny for immediate slaughter and those that sold them as calves was 0.43 standard deviation units and 0.54 standard deviation units, respectively.

**Table 2:** Least squares means (SE in parentheses) predict transmitting ability for direct calving difficulty (Cdiff; percent difficult calvings), gestation length (days), carcass weight (weight; kg), conformation [Conf; scale 1 (poor) to 15 (excellent)], and fat score [Fat; scale 1 (lean) to 15 (fat)] of the sire of the progeny depending on whether the surplus progeny are marketed as calves, yearlings, finishers, or slaughtered immediately on exiting the farm.

	**Beef**					**Dairy**				
**System**	**Cdiff**	**Gestation**	**Weight**	**Conf**	**Fat**	**Cdiff**	**Gestation**	**Weight**	**Conf**	**Fat**
Calves	3.10^a^ (0.06)	0.22^a^ (0.06)	6.29^a^ (0.28)	0.81^a^ (0.02)	0.39^ab^ (0.01)	2.26^a^ (0.02)	−3.57^a^ (0.03)	−6.44^a^ (0.16)	−0.75^a^ (0.01)	−0.30^a^ (0.01)
Yearlings	3.10^a^ (0.09)	0.65^b^ (0.09)	6.73^a^ (0.43)	0.81^a^ (0.03)	0.44^a^ (0.02)	2.30^ac^ (0.03)	−3.40^bc^ (0.05)	−6.05^a^ (0.23)	−0.63^bc^ (0.01)	−0.25^a^ (0.01)
Finishers	3.40^b^ (0.11)	0.88^b^ (0.11)	8.55^b^ (0.53)	0.95^b^ (0.03)	0.33^c^ (0.03)	2.44^b^ (0.04)	−3.47^ac^ (0.06)	−6.38^a^ (0.29)	−0.62^b^ (0.01)	−0.27^a^ (0.005)
Slaughter	3.44^b^ (0.08)	0.85^b^ (0.08)	8.30^b^ (0.40)	0.92^b^ (0.03)	0.36^bc^ (0.02)	2.37^bc^ (0.03)	−3.19^d^ (0.05)	−5.41^b^ (0.22)	−0.66^c^ (0.01)	−0.28^b^ (0.01)

abc *Differences in letters within columns signify a difference (p < 0.05) in least squares means*.

The frequency of beef sire breed used per system is in Table 3; Angus and Hereford were, by far, the most common breeds used in the dairy herds, although the relative frequency of their use differed by system. The odds of a given beef breed service sire being used within each of the four defined systems is in Table 4; this analysis was just limited to the beef-sired progeny. Angus sires were less commonly used in herds that sold their progeny as finisher cattle relative to the other herd systems. Hereford sires were less common among producers who sold surplus progeny as prime cattle for immediate slaughter vs. those who marketed their surplus progeny younger. The likelihood of a Belgian Blue sire being the beef sire used was ~3 times more likely in herds that sold their surplus progeny as calves vs. those that sold them as yearlings or for finishing, with the likelihood also being greater, although not to the same extent, relative to herds that predominantly sold their surplus progeny for immediate slaughter. The preference for the other Continental beef breed sires was greatest in herds that sold their surplus progeny post-weaning, although the odds ratios were not always different to the odds observed for herds that sold their surplus progeny as calves.

**Table 3:** Frequency of the breed of beef sire used by system.

**Breed**	**Calves %**	**Yearlings %**	**Finishers %**	**Slaughter %**
Angus	51.4	50.4	42.2	55.8
Belgian blue	6.4	3.0	3.2	5.7
Charolais	1.0	1.9	1.3	1.7
Hereford	31.9	31.9	28.9	22.0
Limousin	5.7	7.1	20.9	10.0
Simmental	1.8	4.8	2.7	3.5
Other	1.8	0.9	0.8	1.3

**Table 4:** Odds ratio (95% confidence interval in parentheses) of an Angus, Belgian Blue, Charolais, Hereford, Limousin, or Simmental bull being used for each of the different markets when the dataset was limited to just beef sires.

**System**	**Angus**	**Belgian blue**	**Charolais**	**Hereford**	**Limousin**	**Simmental**
Calves	1^a^	1^a^	1^a^	1^a^	1^a^	1^a^
Yearlings	0.83^a^ (0.65, 1.05)	0.32^b^ (0.25, 0.41)	1.36^ab^ (0.99, 1.86)	1.11^a^ (0.88, 1.40)	1.21^a^ (0.94, 1.55)	1.79^b^ (1.35, 2.39)
Finishers	0.46^b^ (0.33, 0.63)	0.40^b^ (0.30, 0.54)	1.24^ab^ (0.81, 1.87)	1.26^a^ (0.93, 1.72)	2.34^b^ (1.71, 3.20)	1.09^ab^ (0.72, 1.64)
Slaughter	1.37^c^ (1.10, 1.69)	0.71^c^ (0.59, 0.86)	1.73^b^ (1.32, 2.26)	0.43^b^ (0.35, 0.53)	1.76^b^ (1.42, 2.19)	1.36^b^ (1.04, 1.79)

abc *Differences in letters within columns signify a difference (p < 0.05) in odds ratios*.

### Dairy Service Sires Used

The difference in mean PTA of the dairy service sires used across herd systems was not as obvious as the trends observed for the beef sires (Table 2). Nonetheless, differences (*p* < 0.05) in genetic merit still existed between the dairy sires used in herds that sold their surplus progeny for immediate slaughter as prime cattle and those that sold their surplus progeny as calves; relative to the latter group of herds, the dairy sires used in herds that sold their progeny as prime cattle for immediate slaughter were genetically more predisposed to a difficult calving with expected longer gestations but were also expected to produce heavier, more-conformed carcasses. The biological differences, however, between the mean PTA of the dairy sires used to produce calves for sale or prime cattle for immediate slaughter were small—for example, a 0.11 percentage unit greater incidence of calving difficulty, a 0.38 day longer gestation PTA, and a 1.03 kg heavier carcass weight PTA. Based on the standard deviation in PTAs of the dairy sires used across the entire dataset, the difference in PTA for carcass weight and conformation of the dairy sires used in the herds that sold their surplus progeny as calves vs. those that sold them for immediate slaughter as prime cattle was 0.15 standard deviation units and 0.27 standard deviation units, respectively.

### Dams

Table 5 summarizes the mean genetic merit for calving performance and carcass merit of the dams of surplus progeny (i.e., cows). Relative to herds that sold the majority of their surplus progeny as calves, the cows in herds that predominantly sold their surplus post-weaning had a greater genetic predisposition to calving dystocia (both direct and maternal) as well as having heavier, more conformed, and fatter carcasses. Relative to herds that sold their surplus progeny for finishing in another premises, the cows in herds that sold their surplus progeny for immediate slaughter as prime cattle were genetically heavier with poorer conformation; although statistically significant (*p* < 0.05), the differences, however, between these two systems were negligible (i.e., 0.51 kg carcass weight PTA and 0.03 units carcass confirmation PTA). In fact, the differences in mean PTA for all traits between systems were biologically small. Using the standard deviation of the PTAs for cows in the entire dataset, the carcass weight and conformation score of cows in herds that sold their surplus progeny as prime cattle for immediate slaughter were 0.27 and 0.46 standard deviation units greater than herds that predominantly sold their surplus progeny as calves; the corresponding value for direct and maternal calving difficulty was 0.22 and 0.21 standard deviation units, respectively.

**Table 5:** Least squares means (SE in parentheses) predict transmitting ability for direct and maternal calving difficulty (CDdiff; percent difficult calvings), carcass weight (weight; kg), conformation [scale 1 (poor) to 15 (excellent)], and fat score [scale 1 (lean) to 15 (fat)] of the dams of the progeny depending on whether the surplus progeny are marketed as calves, yearlings, finishers, or slaughtered immediately on exiting the farm.

**System**	**Direct CD**	**Maternal CD**	**Weight**	**Conformation**	**Fat**
Calves	2.70^a^ (0.02)	6.57^a^ (0.01)	−5.08^a^ (0.10)	−0.59^a^ (0.01)	−0.17^a^ (0.004)
Yearlings	2.79^b^ (0.02)	6.70^b^ (0.02)	−4.29^b^ (0.13)	−0.45^b^ (0.01)	−0.10^b^ (0.005)
Finishers	2.77^b^ (0.03)	6.66^b^ (0.02)	−4.18^b^ (0.16)	−0.44^b^ (0.01)	−0.10^b^ (0.006)
Slaughter	2.88^c^ (0.02)	6.73^b^ (0.02)	−3.67^c^ (0.13)	−0.47^c^ (0.01)	−0.11^b^ (0.005)

abc *Differences in letters within columns signify a difference (p < 0.05) in least squares means*.

## Discussion

In most jurisdictions, the profitability of dairy production exceeds that of many other agricultural commodities, including beef (9, 17). Dairy producers, like most businesses, try to maximize the return on investment of their capital, which includes land, infrastructure, and staff. Compounded by the requirement for a different set of skills for rearing beef cattle, it is for this reason that most dairy producers concentrate solely on the production of milk from dairy cows. Hence, the vast majority of dairy producers tend to sell surplus progeny, including male dairy-bred progeny and all beef-from-dairy progeny, as young calves. This is consistent with the observed frequency distribution in the present study of the different marketing patterns as defined by when the majority of surplus progeny exit the farm. Because of the edits imposed in the present study, all reported frequencies should, however, not be concluded to be representative of Irish dairy herds, although the trend nationally is similar in broad terms (18). Nonetheless, unlike in many other countries, many Irish dairy farms consist of several (relatively small) parcels of land often separated by considerable distance (9), beyond reasonable for milking cows to travel to and from the milking parlor. Therefore, some (Irish) dairy farmers do tend to rear (some of) their surplus progeny post-weaning. This liquid capital can provide a useful cash injection in periods of poor cash flow while also generating revenue from the land that could not be readily used for lactating cows.

Beef producers desire profitable prime cattle that grow rapidly, are efficient, and have a high value at sale; the latter usually implies heavy, well-conformed animals (19), resulting in heavier high-value primal carcass cuts (20). Genetic merit for heavier, more conformed carcasses of prime animals is, however, genetically correlated with greater expected calving difficulty (2), which erodes profit in dairy herds (21); genetic correlations between carcass traits and (direct) gestation length are near zero (22). Many dairy producers select the service sires to be used based on ease of calving (and short gestation length); the downstream impact is, on average, lighter, less-conformed carcasses (2). The main aim of the present study was to determine whether or not the dairy and beef sire selection process differed among herds that predominantly sold their surplus progeny as young calves vs. those that retained their surplus progeny on-farm post-weaning; sire selection here includes both inter- and intra-breed differences. A supplementary objective focused on the genetic merit of especially carcass traits for the cows in these herds and if these differed among herds depending on when the surplus progeny were sold. Such an exercise has never previously been undertaken, although differences in service sire selection by different population strata such as cow parity or herd size have been previously demonstrated (23). The expectation was that dairy producers who rear their surplus cattle post-weaning may place a greater emphasis on the beef characteristics of both the dairy and beef sires used but also may have to compromise in expected calving dystocia. This expectation stemmed from the fact that the price received for an older animal, especially those sold for finishing or immediate slaughter, may more closely reflect its genetic merit, more so than might be obvious in young calves that, at the time of sale, will not fully display their genetic merit for eventual carcass characteristics; the mean (standard deviation) in age of calves when sold from dairy farms in the present study was 25.3 days (10.7 days). Based on a population of 156,864 slaughtered prime cattle, Connolly et al. (24) documented a mean difference of 0.92 kg carcass weight for every 1 estimated breeding value (EBV) difference in carcass weight of the animal; the expectation was 1 kg. A similarly strong association was reported for carcass conformation in the same population with a regression coefficient of phenotype on EBV of 1.08 units (24). That said, using a population of 602 Irish dairy calves aged between 10 and 42 days when weighed, Dunne et al. (25) reported no association between the EBV for conformation and calf live-weight; the genetic evaluation process and trait definitions was the same as that used by Connolly et al. (24). The regression of calf live-weight on EBV for carcass weight was only 0.19 (25); this equates to differences of just 1.89 kg of live-weight per genetic standard deviation unit change in carcass weight, implying that it would be difficult to detect. Hence, expression of inter-animal differences in genetic merit for carcass traits, especially within breed, would be more visible in older animals.

### Genetic Merit by Herd System

While the hypothesis that dairy producers who rear their surplus progeny use, on average, service sires genetically superior for heavier, more-conformed carcasses was confirmed, the actual biological differences between herd systems were negligible to small at best; indeed, the mean difference in genetic merit between the dairy sires used was smaller than the difference between the beef sires, although this was not unexpected given the fact that dairy sires are predominantly used to generate female replacements. The presence of statistical significance, even with such small biological differences, is simply a function of the very large dataset used in the present study, contributing to a very low standard error of the estimated least squares means. Despite this, the mean carcass and calving difficulty PTAs of the sires used in herds that sold their surplus progeny as calves often did not differ from those that sold their surplus progeny as yearlings. What was also obvious in the present study was the extent of beef and dairy sires that were common across the different systems; few sires, however, were common across all four systems. Nevertheless, this could be more due to the (lack of) availability of divergent AI sires than the desired difference in breeding policies of the respective farmers.

Although the mean sire PTA differences between systems in their respective units of measures were small, when expressed relative to the standard deviation in PTAs of the sires used, the difference for some traits was substantial; for example, while the difference in mean PTA for carcass weight between the beef sires used in herds that sold their surplus progeny for finishing and those that sold them as calves was just 2.3 kg, this equated to 0.43 PTA standard deviation units, with the difference being 0.39 SD units when comparing systems that sold surplus progeny as calves vs. for immediate slaughter. Similarly, while a difference in mean carcass conformation PTA among beef service sires used in herds that sold their surplus progeny as calves vs. for finishing or immediate slaughter was only 0.11–0.14 units, based on a 15-point conformation scale, in reality, 70% of all steers slaughtered in Ireland from beef and dairy herds reside within just 5 points of this scale with 86% within 7 points (https://www.gov.ie/en/collection/bc95b-eu-beef-carcase-classification-scheme/#beef-carcase-classification-figures-2020-periodic; accessed February 2021). Interesting, little difference existed in the mean beef and dairy sire PTA for carcass fat score across the systems, suggesting very little emphasis on fat score in sire selection. This probably reflects farmer attitude to the influence of management (e.g., nutrition and age at slaughter) in dictating the eventual fat score of a carcass but also the fact that, of the three carcass traits investigated, proportionally fewer Irish cattle fail imposed processor thresholds on carcass fat score (26).

The choice of service sires used and the lack of meaningful biological difference among herd systems is likely a function of the lack in variability in these traits among the available beef bulls suitable for use on dairy females (i.e., easy calving). Fogh (27) and Berry et al. (2) both outlined the construction of an index to rank beef bulls for use on dairy females, the latter of which was subsequently validated by Berry and Ring (28); the index included both calving performance and beef performance traits marrying the desires of both the dairy and beef producers. However, to be successful, an effective breeding program is required to achieve sustainable genetic gain in this index and identify beef bulls that transmit superior carcass characteristics without a concomitant increased genetic predisposition to a difficult calving. This is especially important given that the mean PTA of (dairy and beef) sires for calving difficulty used by farmers to produce finishers and animals for immediate slaughter was, on average, worse than that used by farmers who sold their surplus progeny as calves (Table 2); gestation length PTAs of the sires used were also longer for the systems that sold surplus progeny post-weaning. Nonetheless, considerable within-breed differences exist for calving performance and carcass traits (29), implying that a breeding program focused on a total merit index comprising both suites of traits should be successful.

Although genetic merit for the service sires and cows was analyzed separately in the present study, in reality, the progeny itself is a manifestation of the germplasm inherited from both parents. The trend in mean parental carcass trait PTAs by herd system was similar for both the service sires and dams. Based on the sum of each parental PTA by herd system, the mean difference in progeny EBV between herds that sold their surplus beef-sired progeny as calves and those that sold them for immediate slaughter was 3.4 kg carcass weight and 0.23 carcass conformation units; the corresponding values comparing surplus beef-sired progeny sold as calves vs. those sold as finishers was 3.2 kg and 0.30 units, with no difference between systems that sold progeny as either calves or yearlings. Furthermore, while the differences in PTAs of the beef service sires among the different herd systems were small, the differences were even smaller for dairy service sires, which is not unexpected given the low selection pressure applied to beef characteristics in the Irish dairy cow breeding goal (3). Indeed, the poorer average genetic merit for carcass credentials in the dairy sires used relative to the beef sires used corroborates previous controlled studies that compared the beef characteristics of dairy vs. beef or beef-on-dairy cattle (30, 31).

The greater risk of dystocia in younger females (21, 32, 33) suggest that nulliparae are likely to be mated to easier calving service sires; this assertion was verified by Berry et al. (23), who compared the genetic merit of dairy and beef sires used in different parity dairy females, including heifers. As well as reporting that genetically easier dairy and beef calving service sires were mated to heifers than cows, the genetic merit for carcass weight and conformation of these service sires was also inferior. Parity was included in the statistical model of the present study to account for herds differing in parity structure. Nonetheless, the mean difference among the four herd system types in the carcass weight and conformation PTA of beef sires used on heifers vs. cows was quantified separately in the present study. The mean difference in carcass conformation PTA of the beef service sires mated to cows in herds that sold surplus progeny as calves vs. for immediate slaughter was 0.13 units (SED = 0.03) but was only 0.07 (SED = 0.02) when matings to heifers were considered; the respective values for PTA for carcass weight of the beef service sires were 2.32 kg (SED = 0.49 kg) and 1.11 kg (SED = 0.49 kg). Therefore, differences in sire genetic merit between herd systems are greater when based on cow matings as opposed to matings to heifers.

While exploitable within-breed genetic variability in carcass merit is known to exist (29), across-breed differences for carcass merit also exist (20, 29, 34). Because differences in the choice of beef sires existed by herd system (Tables 3, 4), of interest in the present study was if the observed difference in mean sire PTA for carcass weight and conformation between herd systems was due to breed substitution differences or within-breed selection. To investigate this, the breed of beef service sire used was fitted as a fixed effect in the statistical model. Even after adjusting for sire breed, differences (*p* < 0.001) remained in mean beef sire PTA for carcass weight and conformation between herd systems; the differences in mean PTA for carcass weight and conformation of beef service sires in herds that sell their surplus progeny as calves vs. for finishing reduced from 2.26 to 0.65 kg and from 0.14 to 0.03 units, respectively; the corresponding values comparing systems that sold surplus progeny as calves vs. for immediate slaughter was a halving from 2.01 to 1.06 kg for carcass weight and from 0.11 to 0.04 units for carcass conformation. Hence, of the observed difference in PTAs for carcass traits among the herd systems, at least half was due to differences in breed choice, with the remaining being attributable to selection within individual breeds. The observed differences in breed choice among herd types (Tables 3, 4) reflect differences in animal price by breed (35, 36) but also known breed preferences among purchasers of progeny at different ages. Although McHugh et al. (37) presented the relative prices paid for animals of different breeds and ages, the breed effects across ages were not directly comparable since the analysis of calf price data was based solely on calves born in dairy herds, while the documented prices of older animals included a combination of progeny from both dairy and beef herds. The frequency of beef breed usage presented for the different beef breeds in the present study (Table 3) is not a reflection of their frequency of use as service sires in dairy herds—this is discussed elsewhere (38); what it does demonstrate, however, is that Belgian Blue sires are more likely to be used in dairy herds that sell their surplus progeny as calves as opposed to post-weaning. Nonetheless, the actual frequency of usage of Belgian Blue sires in the different systems was still low, varying from 3.0% in herds that sold surplus progeny as yearlings to 6.4% in herds that sold surplus progeny as calves (Table 3); the difference in frequency was largely due to a displacement of other continental sire breeds (i.e., Charolais, Limousin, and Simmental), with Belgian Blues in the latter system. Belgian Blue sire usage in dairy herds is quite common in other populations most notably the Nordic countries; Davis et al. (6) stated that, in Nordic countries in the year 2018, 41% of the beef × dairy calves were sired by Belgian Blue sires, while in Denmark, this figure was 80%. The double muscling phenotype of the Belgian Blue is distinguishable even in calves, thus commanding a greater price (37); while as a breed, the Belgian Blue is, on average, more prone to difficult calvings, considerable variability exists within the breed (29), enabling selection from within the breed, especially for dairy females capable of calving larger, more muscular calves. Nonetheless, the proportion of Belgian Blue sired calves born to dairy cows in Ireland has reduced almost consistently in the past two decades from 7% of dairy cow births in 2002 to 2% in 2018.

Results from this study provide insights into the mindset of dairy producers depending on the market of their surplus animals in that dairy producers who sell their surplus progeny as young calves use, on average, beef and dairy service sires that are expected to produce marginally lighter and less-well-conformed carcasses; this is due to a combination of both within- and across-breed selection. While the notion of dairy farmers retaining their surplus animals for rearing may be somewhat unique to Ireland, the results nonetheless are relevant in jurisdictions where relationships exist between individual dairy and beef producers for the supply of quality calves. Moreover, it demonstrates a willingness by dairy farmers to use bulls with greater carcass credentials in anticipation of a reciprocal reward from the market.

## Data Availability Statement

The data analyzed in this study is subject to the following licenses/restrictions: For research purposes only. Requests to access these datasets should be directed to donagh.berry@teagasc.ie.

## Ethics Statement

Data used in the present study were extracted from the Irish Cattle Breeding Federation (http://www.icbf.com) database. Therefore, it was not necessary to obtain animal care and use committee approval in advance of conducting this study.

## Author Contributions

DB and SR contributed to the concept and planning of the article as well as the data editing and analysis. Both authors contributed to drafting the article or revising it critically for important intellectual content.

## Funding

Funding was obtained from a research grant from Science Foundation Ireland and the Department of Agriculture, Food and Marine, on behalf of the Government of Ireland under Grant 16/RC/3835 (VistaMilk).

## Conflict of Interest

The authors declare that the research was conducted in the absence of any commercial or financial relationships that could be construed as a potential conflict of interest.

## Publisher's Note

All claims expressed in this article are solely those of the authors and do not necessarily represent those of their affiliated organizations, or those of the publisher, the editors and the reviewers. Any product that may be evaluated in this article, or claim that may be made by its manufacturer, is not guaranteed or endorsed by the publisher.
